# Subjective and Objective Assessment of the Preferred Rotational Cervical Spine Position in Infants with an Upper Cervical Spine Dysfunction: A Cross-Sectional Study

**DOI:** 10.3390/children11121515

**Published:** 2024-12-13

**Authors:** Anke Langenfeld, Inga Paravicini, Mette Hobaek Siegenthaler, Martina Wehrli, Melanie Häusler, Torsten Bergander, Petra Schweinhardt

**Affiliations:** 1 Department of Chiropractic Medicine, Balgrist University Hospital and University of Zurich, Forchstrasse 340, 8008 Zurich, Switzerland; 2 Accenture, Fraumünsterstrasse 16, 8001 Zurich, Switzerland

**Keywords:** infants, upper cervical spine, range of motion, outcome measure, inertial measurement unit

## Abstract

Background: We aimed to assess (1) the awareness of parents regarding the cervical rotation preference of their infant and the agreement of the parent, clinician and objective assessments, and (2) the test–retest reliability for objective (measured) rotation, lateral flexion and combined flexion–rotation. Methods: This was a cross-sectional study including 69 infants aged three to six months with upper cervical spine dysfunction, without general health issues or specific cervical spine impairments. No treatment was applied. The primary outcomes were parent and clinician assessments of cervical spine rotation preference. The secondary outcome was the cervical range of motion measured by inertial measurement units (IMUs) at two different timepoints. Spearman correlation was performed for the parent, clinician and objective assessments. IMU data were dichotomized into the preferred and unpreferred sides, and test–retest reliability was assessed (ICC). Results: The mean age of infants was 145 days ± 29.1 days, birth length 49.40 cm ± 2.7 cm, birth weight 3328 g ± 530.9 g and 24 were female. In total, 33 infants were assessed by their parents as right-preferred, 30 as left-preferred and 6 as having no preference. The clinician assessed 38 infants as right-preferred and 31 as left-preferred. The correlation between parents and the clinician was r_s_ = 0.687 (*p* < 0.001), the clinician and the IMU r_s_ = 0.408 (*p* = 0.005) and parents and the IMU r_s_ = 0.301 (*p* = 0.044). The ICC of cervical range of motion measurements ranged from poor to moderate. Conclusions: Clinicians can use the parents’ assessment of cervical spine rotation preference as a foundation for their clinical examination. IMU measurements are difficult in infants, possibly due to their lack of cooperation during measurements. Clinical Trial Registration Number: clinicaltrails.gov (NCT04981782).

## 1. Introduction

Torticollis is described as an asymmetric position of the infant’s neck [1,2]. Underlying causes are manifold and can be either traumatic, congenital or acquired [1,3,4,5]. One possible underlying cause is a dysfunction of the upper cervical spine characterized by restricted rotation [6,7].

The clinical picture of upper cervical spine dysfunction is currently not well-investigated [8]. Restricted upper cervical spine rotation can cause a preferred positioning of the head, which can then lead to the development of a plagiocephalus [8]. Over time, plagiocephalus can have an impact on the musculoskeletal health of the growing infant, e.g., scoliosis, neck pain and temporomandibular disorders, if left untreated [9].

During the assessment for upper cervical spine dysfunction, an infant’s cervical spine range of motion is usually assessed [9], though undergoing such an assessment puts stress on infants [10]. Parents may be the first to notice changes or restrictions in their infant’s cervical spine range of motion [9] and might be able to report a cervical range of motion restriction to the clinician. The parents’ perspective of any cervical range of motion restrictions could thus help to decrease the amount of assessment needed for the development of a diagnosis in infants with upper cervical spine dysfunction.

However, a combination of subjective and objective measurements is important to give a more complete overview on the infant’s problem [11]. Inertial measurement units (IMUs) are a valid, reliable and objective outcome measure used for movement analysis, including the cervical range of motion in asymptomatic adults and children with cerebral palsy (CP) aged 4 to 14 years [12,13,14,15,16,17]. IMUs are easily applied and therefore might be useful measurement tools in infants, too [5,17,18,19].

The aims of the present study were to assess parental awareness of cervical range of motion restrictions in their infant and the agreement among parent, clinician and objective assessments, as well as to evaluate the test–retest reliability for rotation, lateral flexion and flexion–rotation in both directions, as measured using IMUs.

## 2. Materials and Methods

This cross-sectional study included data from 69 infants aged three to six months with upper cervical spine dysfunction without general health issues or specific cervical spine impairments. The age range was set according to routine clinical practice based on the rationale for early treatment to support the development of a symmetrical head shape, as the head grows more rapidly during the first five months of life [20]. This study was conducted at two private practices in the canton of Zurich, Switzerland Examination of infants was conducted by the same clinician working at the two practices specialized in the treatment of infants with upper cervical spine dysfunction. Infants were recruited between October 2021 and October 2023. This study was approved by the ethics committee of the canton of Zurich (BASEC-Nr: 2020—02328/17 November 2020) for studies involving humans and has been registered at clinicaltrails.gov (NCT04981782). All parents received a patient information sheet before signing their informed consent.

### 2.1. Primary Outcome

The primary outcomes were parent and clinician assessments of cervical spine rotation and lateral flexion preference at baseline. The parents were asked if there was any preferred cervical spine rotation of their infant and if they were aware of any lateral flexion the infant was positioned in. The clinician visually judged the position of the infant’s cervical spine at the beginning of the physical examination and noted down their impression on an examination sheet (File S1).

### 2.2. Secondary Outcome

The secondary outcome was the cervical range of motion measured by IMUs (Xsens, Enschede, The Netherlands) at two different timepoints. IMUs are a combination of a gyroscope, magnetometer and accelerometer, which have been shown to provide valid and reliable outcome measures for spinal movements [12,15,16,17]. IMUs are appropriate devices to measure spinal motion as movement can be measured using nine degrees of freedom around one movement axis [14].

### 2.3. Study Procedure

**Figure 1:**
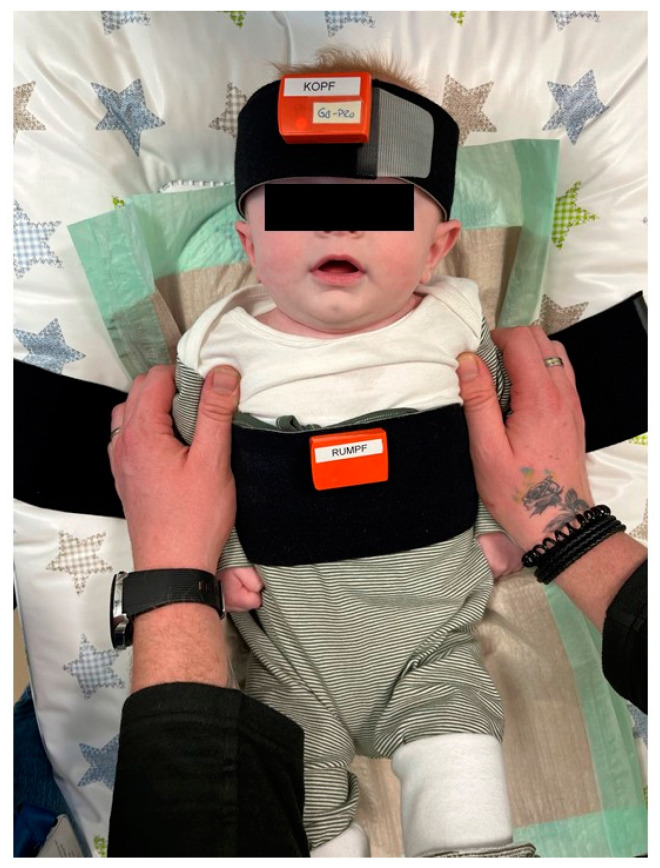
Positioning of the inertial measurement units on the child, positioning of the child and positioning of the parent’s hands on the child during measurement.

Parents were interviewed about the course of pregnancy, such as health issues, child birth, such as the birth mechanism, and previous therapies at baseline. Furthermore, they were interviewed regarding any current issues such as breast-feeding difficulties. Additionally, they were asked for their impression of their infant’s preferred cervical spine rotation and lateral flexion. Afterwards, the first measurement of cervical spine rotation, lateral flexion and flexion–rotation was conducted by an assessor trained in manual therapy. For the measurement, a Velcro strap was put around the infant’s torso and an IMU was attached to it. A second Velcro strap was put around the infant’s head and an IMU was attached in line with the IMU on the torso (Figure 1). To prevent any upper body movement of the infant, parents were asked to put their hands on the infant’s shoulders and fixate the upper body on the treatment table (Figure 1).

Then the infant’s cervical spine was moved by the assessor into rotation, lateral flexion and flexion–rotation using a standardized order of movement. All movements were conducted once. If a child was too unsettled, only flexion–rotation was conducted, because this is the most relevant movement for upper cervical spine dysfunction. Infants were allowed to use a pacifier during measurements. After the first measurement, the assessor left the room and the clinician entered. Now, a physical examination of the infant was conducted by the clinician. During this examination, the clinician also judged the preferred cervical spine rotation and lateral flexion. Afterwards, the assessor entered the room again and the second measurement was conducted following the same procedure as during the first measurement. No treatment was conducted between the two measurements. An additional sample (8 infants/10 adults) was recruited and measured to verify the measurement settings and procedure.

### 2.4. Data Preparation

**Figure 2:**
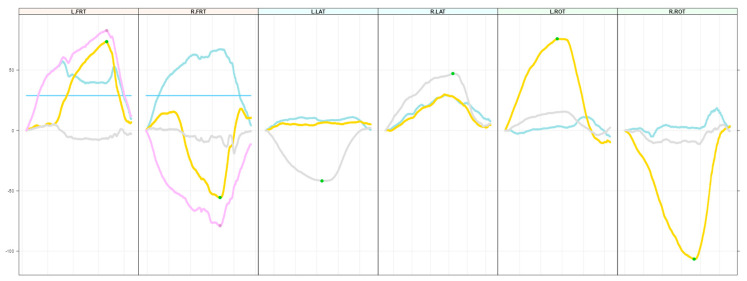
Curves for flexion–rotation test left (L.FRT) and right (R.FRT), lateral flexion test left (L.LAT) and right (R.LAT) and rotation test left (L.ROT) right (R.ROT). For flexion–rotation test, the blue curve represents the flexion of the cervical spine, the yellow curve rotation and the blue straight line the threshold of flexion needed to qualify flexion–rotation as valid. The green dots signify good measurements without flaws. The gray line resembles lateral flexion and the yellow line rotation. The purple line is the combined representation of flexion and rotation, and the point in time with maximum combined (flex/rot) rotation is marked with a purple dot. This point in time is used to obtain rotation degrees (yellow line point). On the yellow line, it is marked as “green” (good) when the flexion degree at this point in time is above the threshold, and otherwise red.

This study used two IMUs. Before each measurement, an alignment reset was performed to zeroize measurements. Data were recorded separately for every movement. Afterwards, raw data were extracted in quaternions. Quaternions are an extension of complex numbers and are frequently used to illustrate four-dimensional movements [21]. They do not suffer from gimbal lock during combined movements such as flexion–rotation [21]. Quaternions were transformed in degrees of cervical range of motion using R (Version 4.3.2) [22]. Subsequently, all data were illustrated in figures (Figure 2) representing time-courses of the movements. Figures were visually inspected by TB and AL for plausibility.

### 2.5. Statistical Analysis

Study data were managed using the REDCap Electronic Data Capture system [23,24]. Statistical analysis was performed using SPSS (Version 29, IBM, Armonk, NY, USA). Demographics were determined and descriptive statistics of the sample were calculated. To assess the correlation between parent, clinician and IMU data, Spearman correlation was performed [25]. IMU data were dichotomized into preferred and unpreferred sides, according to the torticollis side determined by the clinician, and the intraclass correlation coefficient (ICC) was calculated (test–retest). The ICC model used was two-way mixed effects, type: mean of k measurements and definition of relationship: absolute agreement [26]. Values less than 0.5 represent fair agreement, between 0.5 and 0.75 moderate, between 0.75 and 0.9 good and above 0.9 excellent agreement [26]. Furthermore, the standard error of measurement (SEM) (SEM = SD $\sqrt{1-ICC}$) [27] and minimal detectable difference (MDD) (MDD = 1.96 ∗ $\sqrt{2}*SE$) were calculated [28] to assess absolute reliability.

## 3. Results

### 3.1. Demographics and Descriptive Statistics

Sixty-nine infants (mean age at enrollment 145.31 (SD 29.09) days, birth weight 3328.74 (SD 530.94) grams and birth length 49.40 (SD 2.75) cm) were included. Of these, 45 were male and 24 females. Three infants were born before gestation week 36. Two infants were diagnosed with hip dysplasia (one left, one right). Five infants had foot anomalies, e.g., six toes, and none had a club foot. At baseline, 58 infants were described by their parents as having a head asymmetry. Nineteen infants had no previous therapy for torticollis. Fifty infants had received previous treatment such as physical therapy, osteopathy, positioning or a combination of therapies. Six infants had older siblings who experienced the same cervical spine issues during infancy.

Thirty-five mothers were primipara and thirty-four were multipara (two to four pregnancies). Sixty infants had a cephalic presentation, three were in the breech position and one was in the transverse position. Thirty-nine infants were born vaginally, in three cases an additional episiotomy was needed, six infants were born using vacuum extraction and twenty-one were born via a cesarean section due to infantile reasons such as a decreased heart rate or maternal reasons such as fever. Thirty-two mothers were breastfeeding, of which seven had experienced a problem with either the left (five) or the right (two) side. Thirty-two mothers were not breastfeeding. Three mothers had birthed twins. Fifty-one mothers did not experience any problem during delivery, while eighteen mothers experienced problems during delivery such as a decreased heartbeat of the infant combined with a subsequent emergency cesarean section.

Six children were rated by their parents as having no rotational preference. Six had a fixed rotation to either the left (three) or right (three) side. Thirty had a preferred rotation to the right but could rotate to the left, and twenty-seven had a preferred rotation to the left but could rotate to the right. Regarding lateral flexion, 29 infants were rated by their parents as not having any preferred lateral flexion. Three were rated as having fixed lateral flexion to the right and four to the left. Thirteen were rated as having a lateral flexion to the right but moving to the left was possible, and seventeen were rated as having preferred lateral flexion to the left but moving to the left was possible. The clinician rated 31 infants as having a rotational preference to the left, and 38 were rated as having a rotational preference to the right. Lateral flexion was rated as preferred to the left in 38 infants and to the right in 31 infants.

### 3.2. Subjective and Objective Assessments

Correlation of the subjective assessment of preferred rotation between parents and the clinician was high (r_s_ = 0.687, *p* < 0.001). Correlation of parents’ subjective assessment of preferred rotation and the objective assessment was moderate (r_s_ = 0.408, *p* = 0.005), as well as the correlation between the clinician subjective assessment and the objective assessment (r_s_ = 0.301, *p* = 0.044). These correlations did not change when infants were dichotomized as “previous treatment” and “no previous treatment”. No correlation was found between any assessments of lateral flexion, e.g., correlation of parents and the clinician (r_s_ = 0.045, *p* = 0.720).

### 3.3. Test–Retest Reliability

The ICC for rotation was poor for the preferred (0.352, 95%CI −0.597–0.737) and unpreferred (0.462, 95% CI −0.256–0.766) sides. The SEM for the preferred side was 10.01° and the MDD was 27.71°, and the SEM for the unpreferred side was 7.60° and the MDD was 21.06°. The ICC for lateral flexion was poor for the preferred (0.076, 95% CI −1.159–0.594) and moderate for the unpreferred (0.677, 95% CI 0.293–0.852) side. The SEM for the preferred side was 8.83° and the MDD was 24.46°, and the SEM for the unpreferred side was 5.12° and the MDD was 14.18°. The ICC for flexion–rotation was poor for the preferred (0.471, 95% CI 0.024–0.713) and unpreferred (0.350, 95% CI −0.122–0.643) sides. The SEM for the preferred side was 11.07° and the MDD was 30.65°, and the SEM for the unpreferred side was 11.96° and the MDD was 33.13°.

## 4. Discussion

This study found a high correlation between parents’ and the clinician’s assessments of the preferred direction of cervical spine rotation and a moderate correlation for parents’, clinician’s and the objective assessment of the preferred direction of cervical spine rotation. The ICCs for rotation, lateral flexion and flexion–rotation were poor to moderate, while the SEM and the MDD were large for most movement directions.

The high correlation of the preferred direction of cervical spine rotation for parents and the clinician indicates that parents’ judgement is valuable for the assessment of infants with an upper cervical spine dysfunction. Therefore, parents’ judgment regarding possible malpositioning of the cervical spine may support the clinician’s findings during examination of the infant. Furthermore, using multiple outcome measures helps to increase the scientific rigor in research with infants [11]. A recent scoping review by Hayton et al. recommends either using a performance-based outcome measure (Alberta Infant Motor Scale) or clinician-reported outcome measures (latch, audible swallowing, type of nipple, comfort, hold (LATCH)) to evaluate the efficacy of spinal mobilization or manipulation for a variety of medical conditions in infants [29]. However, these assessment tools all have a subjective bias as they are either reported by the treating clinician or an observer. In particular, musculoskeletal research in infants would benefit from valid, reliable and responsive objective outcome measures with reduced subjective bias. IIMUs have been shown in different clinical settings to provide reliable and valid measurements and in adults and in children aged between two months and fourteen years [12,13,14,15,16,17,18,19]. It was therefore unexpected that that the ICC ranged between poor and moderate for all movement directions in the present study. Factors that might have contributed to this poor outcome are a lack of fixation of the shoulders of the infant by the parents and the high number of measurements and directions tested. To test these two factors, the additional sample of eight infants was measured only for flexion–rotation and with parents replaced with an experienced pediatric clinician who fixated the infant. The ICC remained moderate for flexion–rotation (preferred side: 0.471, 95% CI 0.024–0.731, unpreferred side: 0.350, 95% CI −0.122–0.634). Next, the passive range of motion of the cervical spine was measured in a group of ten adults using the same IMUs and same set-up as for the infants. Here, the ICC was excellent (>0.9). This confirms that assessment of spinal movements using IMUs is valid and reliable. Thus, the most likely explanation for the poor quality of the IMU measurements in the current study is the lack of cooperation of the infants during the measurements. In general, infants are a special group of participants. They are very sensitive to their environment and external stimulation and do not yet have the capability to cooperate willingly [11]. Furthermore, the emotions of the parents, especially the mother, can be transferred to the infant [30]. Cooperation of the infant usually starts during the second year of life [31]. Of note, the studies cited above that used IMUs successfully in infants assessed spontaneous active movements [26,27], meaning they did not rely on the cooperation of the infants. Another study [17] investigated instructed active movements, thus relying on the children’s collaboration, but here, the children were between 4 and 14 years of age. One study that attempted to measure the passive cervical range of motion in infants [5], similar to the present study, found ICC values very similar to the ones found here. From these studies and the present one, it can be concluded that the poor to moderate measurement reliability in infants stems from attempting to measure passive movements. As in other studies on infants, spontaneous active movements might offer a valuable alternative for the assessment of upper cervical spine dysfunction.

The rationale for early treatment, starting at the age of three months, is to support the development of a symmetrical head shape, as the head grows more rapidly during the first five months of life [20]. 

The findings of this study should be interpreted in light of one limitation. All infants included in this study were seen by the same clinician. Therefore, personal interference between the clinician, parents and infant could have influenced the outcomes.

## 5. Conclusions

This is one of the first studies to evaluate IMU use in infants, especially in infants with upper cervical spine dysfunction. Our findings highlight (i) the challenges of using objective outcome measures in infants, and (ii) that parents are capable of judging the cervical spine rotational position of their infant well and in line with a clinician’s judgment. For future sensor-based research protocols, tools should be developed that are more tailored to the needs and capabilities of infants, to guarantee valid and reliable research results.

## Data Availability

The raw data supporting the conclusions of this article will be made available by the authors on request.

## Supplementary Materials

The following supporting information can be downloaded at https://www.mdpi.com/article/10.3390/children11121515/s1, File S1.
