# Supplementary material for: Subjective and Objective Assessment of the Preferred Rotational Cervical Spine Position in Infants with an Upper Cervical Spine Dysfunction: A Cross-Sectional Study
Source: Children (Basel). 2024 Dec 13;11(12):1515. doi: 10.3390/children11121515 (PMC11675051; doi:10.3390/children11121515)
Supplement: Supplementary file 1 [file children-11-01515-s001.zip › children-3336882-supplementary.pdf]

Examination

Record ID	__________________________________
Rotational malposition of the cervical spine in supine position	◯ left<br>◯ right<br>◯ no malposition<br>◯ n/a
Lateral tilt of the cervical spine in supine position	◯ left<br>◯ right<br>◯ no tilt<br>◯ n/a
Head asymmetry?	◯ Plagiocephalus right<br>◯ Plagiocephalus left<br>◯ Brachycephalus<br>◯ Brachy-Plagiocephalus right<br>◯ Brachy-Plagiocephalus left<br>◯ None
Amount of asymmetry	◯ mild<br>◯ moderat<br>◯ severe

Posterior Flattening Assessment

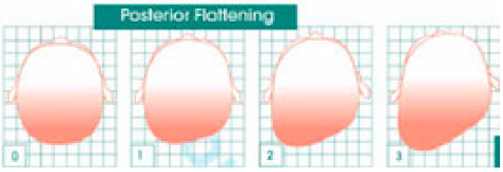

Posterior Flattening Score	◯ 0<br>◯ 1<br>◯ 2<br>◯ 3

Facial Asymmetry Assessment

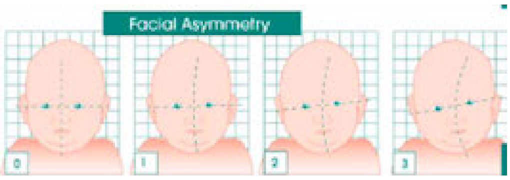

Facial Asymmetry Score	◯ 0<br>◯ 1<br>◯ 2<br>◯ 3

Forehead Asymmetry Assessment

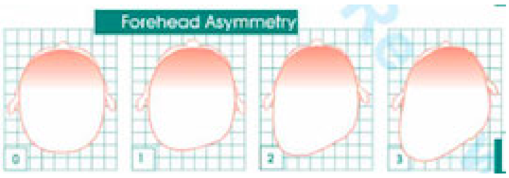

Forehead Asymmetry Score

- ◯ 0
- ◯ 1
- ◯ 2
- ◯ 3

Ear Misalignment Assessment

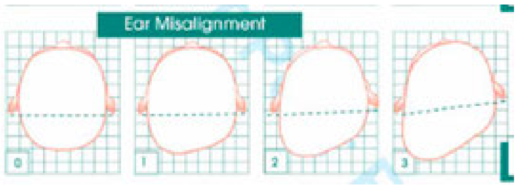

Ear Misalignment Score

- ◯ 0
- ◯ 1
- ◯ 2
- ◯ 3

Measurement calliper (left)

____
(Measure from left eyebrow to right occiput)

Measurement calliper (right)

____
(Measure from right eyebrow to left occiput)

Pull-to-sit (head centered)

- ◯ yes
- ◯ no
- ◯ barely
- ◯ n/a

Active rotation in supine position

- ◯ complete
- ◯ incomplete left
- ◯ incomplete right
- ◯ incomplete left and right

Compensation with opposite shoulder movement in supine position

- ◯ yes
- ◯ no
- ◯ n/a

Sitting

- ◯ hold
- ◯ free

Active rotation in sitting position

- ◯ complete
- ◯ incomplete left
- ◯ incomplete right
- ◯ incomplete left and right

Compensation with opposite shoulder movement in sitting position	◯ yes<br>◯ no<br>◯ n/a
Lifting head in prone position possible	◯ possible<br>◯ impossible<br>◯ barely possible<br>◯ n/a
Arm position during head lifting	◯ no support<br>◯ elbow support<br>◯ hand support
Paravertebral muscles normotonic	◯ yes<br>◯ no<br>◯ n/a
Lateral neck muscles normotonic	◯ yes<br>◯ no<br>◯ n/a
Palpation SCM muscle	◯ Yes<br>◯ No
Finding during palpation of SCM muscle	◯ symmetrical<br>◯ asymmetrical<br>◯ n/a
SCM irregularity	◯ fibrosis right<br>◯ fibrosis left<br>◯ pseudotumor right<br>◯ pseudotumor left<br>◯ hypertension right<br>◯ hypertension left<br>◯ other irregularities
Other irregularities	______________________________
Other parts of the spine, apart of the c-spine, restricted ?	◯ yes<br>◯ no<br>◯ n/a
Which part?	☐ BWS<br>☐ LWS
Sacroilical joint in examination	◯ normal<br>◯ abnormal
Resistance to hip abduction	◯ yes<br>◯ no<br>◯ n/a
Was there any additional examination of the cervical soft tissue?	◯ Yes<br>◯ No

Sonography	◯ Yes<br>◯ No
Finding Sonography	__________________
Diagnosis	__________________
[redacted]	[redacted]
[redacted]	◯ [redacted]<br>◯ [redacted]<br>◯ [redacted]<br>◯ [redacted]
